# Supplementary material for: Single-Cell Transcriptomics Reveals the Complexity of the Tumor Microenvironment of Treatment-Naive Osteosarcoma
Source: Front Oncol. 2021 Jul 21;11:709210. doi: 10.3389/fonc.2021.709210 (PMC8335545; doi:10.3389/fonc.2021.709210)
Supplement: Supplementary Document 1 — Kaplan-Meier curve of the 518 genes associated with survival. [file DataSheet_1.zip › Supplementary Document 1/sur.TRIM21.pdf]

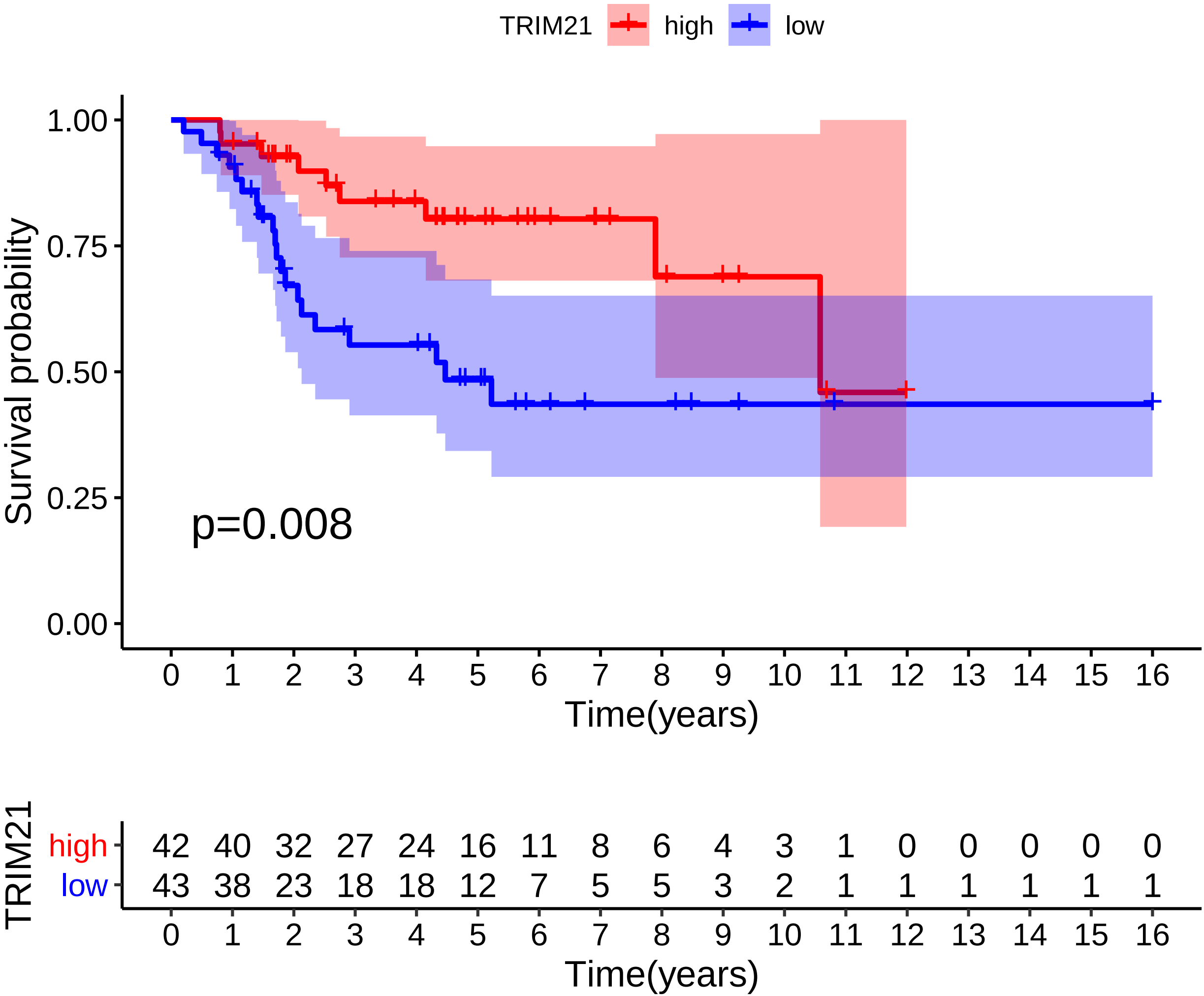
TRIM21
high
low
1.00
0.75
0.50
0.25
0.00
Survival probability
p=0.008
0 1 2 3 4 5 6 7 8 9 10 11 12 13 14 15 16
Time(years)
TRIM21
high 42 40 32 27 24 16 11 8 6 4 3 1 0 0 0 0 0
low 43 38 23 18 18 12 7 5 5 3 2 1 1 1 1 1 1
0 1 2 3 4 5 6 7 8 9 10 11 12 13 14 15 16
Time(years)
